# Physical Activity, Mental Health and Consumption of Medications in Pre-Elderly People: The National Health Survey 2017

**DOI:** 10.3390/ijerph18031100

**Published:** 2021-01-26

**Authors:** Juan Manuel Carmona-Torres, Ana Isabel Cobo-Cuenca, Diana P. Pozuelo-Carrascosa, Pedro Ángel Latorre-Román, Juan Antonio Párraga-Montilla, José Alberto Laredo-Aguilera

**Affiliations:** 1 Facultad de Fisioterapia y Enfermería, Campus de Fábrica de Armas, Universidad de Castilla-La Mancha, Av de Carlos III, nº 21, 45071 Toledo, Spain; juanmanuel.carmona@uclm.es (J.M.C.-T.); anaisabel.cobo@uclm.es (A.I.C.-C.); JoseAlberto.Laredo@uclm.es (J.A.L.-A.); 2 Grupo de Investigación Multidisciplinar en Cuidados (IMCU), Campus de Fábrica de Armas, Universidad de Castilla-La Mancha, Av de Carlos III, nº 21, 45071 Toledo, Spain; 3 Departamento de Didáctica de la Expresión Musical, Plástica y Corporal, Universidad de Jaén, Campus Las Lagunillas s/n, 23071 Jaén, Spain; platorre@ujaen.es (P.Á.L.-R.); jparraga@ujaen.es (J.A.P.-M.)

**Keywords:** physical activity, depression, mental health, medications, active aging, Spain

## Abstract

*Background***:** The promotion of Physical Activity (PA) is an important public health goal to reduce comorbidity and diseases associated with aging such as anxiety and depression. *Aim***:** To investigate the association between level of PA, mental health and the consumption of medications among a representative cohort of Spanish pre-elderly people. *Methods***:** Cross-sectional study with 5977 participants aged 50 to 64 years who participated in the National Health Survey in Spain 2017. The levels of PA were evaluated using the International Physical Activity Questionnaire and the mental conditions were measured by the Goldberg Health Questionnaire-12 (GHQ-12). The chi-square test was used for qualitative variables; Pearson’s correlation was conducted between GHQ-12 score with different quantitative variables; and a logistic regression was used to determine the association between PA and mental health with the sociodemographic characteristics. *Results***:** The participants were 51.9% women and 48.1% men with a mean age of 56.79 years, and 35.5% of participants had a low level of PA. A low level of PA was associated with cases of mental health vulnerability, anxiety and depression (in women), the consumption of more medications and greater multimorbidity. *Conclusion***:** It is important that people reach old age with an optimal health status in order to reduce age-related disability and morbidity. More than a third of the Spanish pre-elderly do not reach the levels of PA recommended by the WHO. People who had low level of PA consumed more medications and had higher mental health vulnerability and greater multimorbidity.

## 1. Introduction

The world’s population is aging faster than in recent decades [1,2]. In fact, 1 in 6 people in the world will be over 65 in 2050. In Spain, 25% of the population will be over 65 in 2030 and it will be the European country with the largest increase in the share of older persons by 2050 [1]. For this reason, increasing numbers of older adults are living with disabilities and health conditions that are preventable through lifestyle modifications [3]. For this, active aging is a very important issue for enabling individuals to reach retirement in the best possible health condition in this aging society.

Population aging is a public health success story, but at the same time it represents the challenge of maintaining the quality of life, functional capacity, and social participation of older adults [4]. In fact, there is an increasing incidence of mental disorders in the elderly, with depression and anxiety being the most commonly diagnosed mental disorders [5,6]. The World Health Organization (WHO) estimates that approximately 1 in 10 older adults may suffer from depression, with the prevalence increasing with age [7,8]. Depression is commonly treated with antidepressants in primary care. However, antidepressants can have adverse side effects, low treatment adherence, and there is a delay between the beginning of antidepressant use and improvements in mood [9]. In addition, the use of antidepressant medication is associated with recurrent falls in elderly individuals [7,10]. In the case of people with anxiety disorders, antidepressant drugs such as selective serotonin reuptake inhibitors (SSRIs) are the first-line treatments. However, one-third of patients do not respond to SSRIs [11]. Considering that depression is currently the leading cause of the disability burden worldwide and that its prevalence increases with age [12], it is necessary to act on the modifiable risk factors that are related to it, such as physical inactivity [13].

The aging process associated with sedentary lifestyles promotes mental, social and physical dependence, and is associated with all-cause mortality and premature death [6,14]. In fact, physical inactivity is the fourth leading risk factor for global mortality [15]. In contrast, there are several studies that have shown that physical activity (PA) protects from the emergence of depression and anxiety [9,16,17]. PA is a key determinant of physical, mental and social health in all age groups and it associated with improved health status [18]. Promoting and maintaining higher levels of PA in the older population is an imperative for active aging [3]. Achieving >150 min/week of moderate-intense aerobic exercise is associated with at least a 30% lower risk of morbidity, mortality, and functional dependence compared with being inactive [19]. Moreover, PA prevents and reduces the risk of osteoporosis, type 2 diabetes, hypertension obesity, stroke, heart disease, colon cancer, breast cancer and depression [3,15,20,21]. In addition, regularly performed PA is one of the main non-pharmacological strategies for healthier aging and is associated with the lower consumption of medications [22]. It is noteworthy that in the last decade in Spain, older people have decreased their levels of PA while the consumption of medications has increased [21,23]. In fact, recent studies show that people who do PA several times a week consume almost 20% less medication than people who never do PA [24].

The PA should be a common habit with the aim of having healthy aging and reaching retirement age with the best health and quality of life. A recent study in Poland suggests that physical inactivity begins at age 50 [25]. At this age, women also begin menopause, which can lead to a loss of aerobic fitness, muscle strength, and bone mineral density, as well as weight gain [26]. This increases the risk of many chronic diseases such as coronary heart disease, type 2 diabetes mellitus or osteoporotic fractures, especially in sedentary individuals [27]. To counteract the effects of this stage of life, PA plays an important role because it improves muscle strength, increases bone metabolism and functional capacity, prevents type 2 diabetes and decreases obesity and depression, thereby improving the quality of life and enabling people to reach old age with better health in general [21,26]. As a result, PA is an important public health objective to mitigate the burden of age-related diseases [28].

In Spain, there are programs for older people to promote PA with the aim of guaranteeing the Spanish population universal access to PA, reducing sedentarism and obesity and to promote healthy lifestyle habits [29]. In addition, these PA promotion programs also aim to improve mental health in the Spanish population. However, PA levels have decreased from 2009 to 2017, with an average of 1.32 h per week spent on moderate PA and 1.28 h per week on walking [21].

Therefore, PA could be an effective intervention both to improve mental health (mainly to reduce anxiety and depression) and to reduce the consumption of medications (including that associated with mental disorders as antidepressants, benzodiazepines, etc.). For this reason, it is important to evaluate the association between PA levels and anxiety and depression, because these aspects can slow down the symptoms associated with the prevalence of chronic degenerative diseases [6]. Although there are many studies about the benefits of PA in mental health in older people [5,6,9,16,17], to the best of our knowledge, there are no known studies that have been conducted on pre-elderly people (a stage that ranges from 50 to 64 years according to several authors [30,31,32]) that relates mental health to levels of PA and the consumption of medications. Our hypothesis is that pre-elderly people who do moderate or intense PA have better mental health (suffer fewer problems of depression and anxiety), less consumption of medications and have a better state of health than sedentary people.

Based on the afore, the objectives of this study were: (1) to investigate the association between PA levels, mental health and the consumption of medications among a representative cohort of Spanish pre-elderly people; (2) to know the prevalence and associated factors of level of PA in Spanish pre-elderly people.

## 2. Materials and Methods

### 2.1. Study Design and Participants

This is a cross-sectional study, performed with secondary data from the National Health Survey in Spain (NHSS) 2017 [33]. The NHSS was carried out through a personal interview by the National Statistics Institute (Instituto Nacional de Estadística—INE) and the Ministry of Health, Social Services and Equality (Ministerio de Salud, Servicios Sociales e Igualdad—MSSI). The NHSS use probabilistic multistage sampling with stratification of the first-stage units (census sections) and the second-stage units (main family dwellings), with the final units (individuals) being selected by means of random routes and sex- and age-based quotas. These data from the NHSS are available as anonymized data on the INE web. As we used public anonymized secondary data, the approval of an ethics committee was not necessary, according to Spanish legislation.

For the purpose of the current study, the study population was restricted to non-institutionalized individuals between 50 and 64 years of age, which is the pre-elderly stage age according several studies [30,31,32]. Thus, the number of participants included in the study was 5977.

### 2.2. Instruments and Variables

The data collection instruments used by the INE and MSSI, in a transverse way, was 2017 EHSS [33]. In this survey, participants are asked about the following variables:

1. Sociodemographic variables: age, sex, marital status, level of education and social class (according to Domingo-Salvany et al. [34]: class I: directors and managers of companies with 10 or more employees and professionals with university degrees; class II: directors of companies with fewer than 10 employees and professionals with college diplomas; class III, intermediate occupations; class IV: workers in qualified technical occupations; class V: primary sector workers; and class VI: unskilled workers).
2. Mental health variables: self-reported depression and anxiety in the last 12 months (with a medical diagnosis) and mental conditions (psychological morbidity and possible cases of non-psychotic psychiatric disorders).
3. Health-related variables such as health self-perception, disease or chronic/long-term illnesses, multimorbidity (according to the WHO [35], multimorbidity is considered the coexistence of two or more chronic conditions in the same individual, and data were collected across 32 medical diagnoses), body mass index (BMI) (according to WHO criteria [36], BMI is categorised as follows:<18.5 kg/m^2^, underweight; 18.5–24.9 kg/m^2^, normal; 25.0–29.9 kg/m^2^, overweight; and ≥30 kg/m^2^, obese), the consumption of medications in the last 2 weeks (including the consumption of tranquilisers/relaxants/sleeping pills and the consumption of antidepressants/stimulants) and polypharmacy (according to the WHO [37], polypharmacy is considered the simultaneous consumption of 5 or more medications; our study referred to the last two weeks).
4. Physical Activity variables: type of PA performed in the main activity, frequency with which they perform PA, PA performed in the last 7 days (intense/moderate), number of days a week in which they walk for at least 10 min, time spent walking per week and time spent doing physical exercise in leisure time.

The mental conditions were measured by the Goldberg Health Questionnaire-12 (GHQ-12), Spanish version [38], which is a self-administered screening instrument aimed at detecting psychological morbidity and possible cases of non-psychotic psychiatric disorders. The questionnaire consists of 12 items, and response options range from 0 (never) to 3 (more than usual). We transformed the typical Likert scale (0-1-2-3) into a dichotomy punctuation (0-0-1-1) in order to obtain a 12-point scale variable because the literature suggests that this is the most appropriate score [38,39]. In addition, we chose a cut-off score greater than 5 to detect cases of mental health vulnerability in the participants (GHQ-12 ≤ 5 = non case and GHQ-12 ≥ 6 = case) [40].

PA was measured by the short version of the International Physical Activity Questionnaire (IPAQ), Spanish version [41]. The IPAQ consists of 7 self-reported questions that assess the intensity (low, moderate or vigorous), frequency (days and hours per week) and duration or time spent on each of the activities. PA was defined as the level of self-reported engagement in moderate activity in a typical week: both days/week and h/week and walking for 10 min (days/week). Moderate activities were those that produce a slightly stronger than normal increase in breathing, heart rate and sweating for at least 10 min in a row, and may include carrying light weights, riding a bicycle at normal speed, participating in sports, or gardening. Vigorous activities were those that produced a much greater increase than the previous one in the same variables for at least 10 min [41,42,43]. Based on previous studies, weekly PA was measured in METs (unit of measurement for metabolic rate)-min per week (MMS) [41,44]. The MMS was calculated by multiplying the baseline MET values according to intensity (walking = 3.3 METs, moderate PA = 4 METs and vigorous PA = 8 METs) by the minutes and days spent walking, moderate PA and vigorous PA, i.e., MET-min per week: MET level x minutes of activity x events per week. Once these values were obtained, they were added together to find the total PA performed. According to the results, the participants were distributed into 3 activity categories [41,42,43]:

1. Low: Participants who did not register activity or did not meet the criteria for moderate and high categories.
2. Moderate: Participants who met one of these criteria:
  - 3 or more days of vigorous PA for at least 20 min/day
  - 5 or more days of moderate PA or walking for at least 30 min
  - 5 or more days of any combination of walking, moderate or vigorous PA, achieving at least 600 MMS.
3. High: Participants who met one of the following criteria:
  - 3 or more days of vigorous PA or accumulating at least 1500 MMS
  - 7 or more days of any combination of walking, moderate or vigorous PA, achieving a minimum of at least 3000 MMS.

### 2.3. Statistical Analysis

We calculated descriptive measures for all variables of interest by calculating counts (n) and proportions (%) for qualitative variables and by calculating means (m) and standard deviation (SD) for quantitative variables. The Kolmogorov–Smirnov test was used to test for data normality. The chi-square test (χ^2^) was used to compare categorical variables between groups. For quantitative variables, a Pearson’s correlation analysis (r) was performed. To control for the influence of sex and age, partial correlations were performed. The consumption of medications levels threshold that best discriminated the mental health vulnerability, depression, anxiety and multimorbidity was determined by using the receiver operating characteristic (ROC) curve. We generated multivariable logistic regression models to assess factors independently associated with a low level of PA. The Wald statistic was used, in which variables with *p* ≥ 0.15 were eliminated from the model. Unadjusted and adjusted odds ratios (ORs) were calculated with 95% confidence intervals. In addition, sex-stratified multivariable logistic regression models for low level of PA were calculated. All of the contrasts of hypotheses were bilateral and statistical significance was established at *p* < 0.05.

Data were analyzed using IBM SPSS Statistic v.24.0 (IBM Corp. Armonk, NY, USA), licensed by the University of Castilla La-Mancha.

## 3. Results

The total sample was 5977 pre-elderly (age between 50 and 64 years old), including 51.9% women and 48.1% men, with a mean age of 56.79 years (SD ± 4.31). Table 1 shows the characteristics of the sample by sex. Most of the participants were married (66.4%), had primary education (47.6%), and belonged to social class V (32.3%). The majority had no mental disease with an average score of 1.48 (SD ± 2.74) in GHQ-12. Overall, 51.8% of pre-elderly people perceived their health status as good, although 74.6% had a disease or chronic/long-term illness, 61.4% morbidity and 41.5 were overweight. The mean medication consumption was 1.84 (SD ± 1.89) and 10.1% present polypharmacy (≥5 medications consumed in the last 2 weeks). In total, 44.1% had a moderate level of PA.

**Figure 1:**
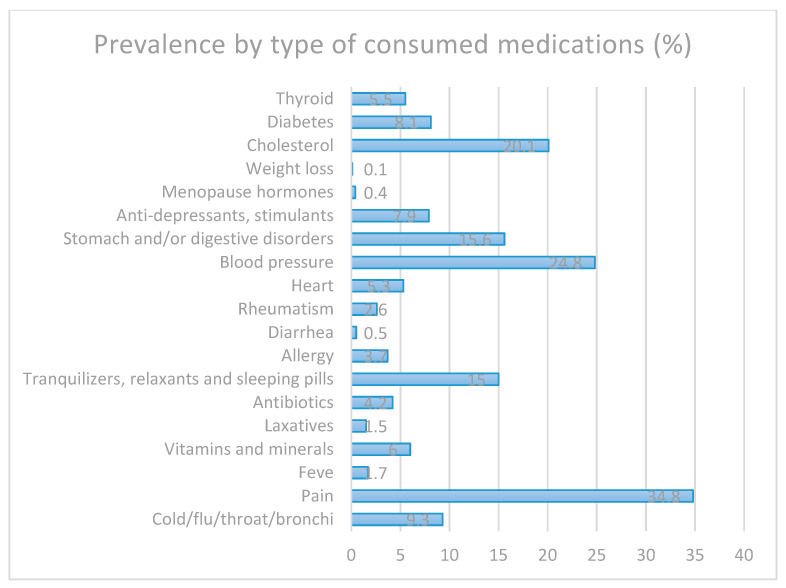
Prevalence of the different groups of medications consumed by the Spanish pre-elderly.

The prevalence of the different groups of medications consumed in the last 2 weeks by the pre-elderly is shown in Figure 1. The most commonly consumed medications in the Spanish pre-elderly were those for pain (34.8%) blood pressure (24.8%) and cholesterol (20.1%). Regarding the consumption of medicines for possible mental disorders, 15% consumed tranquilizers, relaxants or sleeping pills and 7.9% consumed anti-depressants.

**Figure 2:**
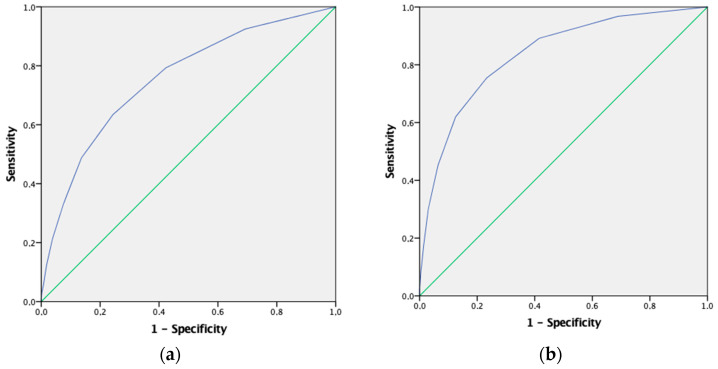

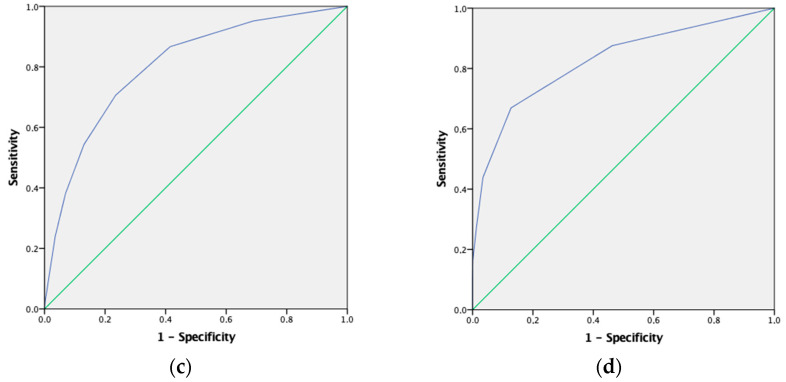
ROC curve that summarizes the potential of the number of medications consumed in the last 2 weeks to identify cases of mental health vulnerability (**a**) depression in the last 12 months (**b**), anxiety in the last 12 months (**c**) and multimorbidity (**d**).

Pre-elderly individuals who had low levels of PA consumed more drugs, as did people who had a case of mental health vulnerability or multimorbidity (*p* < 0.001). Figure 2 shows the ROC curve for cases of mental health vulnerability (Figure 2a) [area under the curve (AUC) = 0.775, 95% CI = 0.734–0.776; *p* < 0.001, the cut-off point was 1.5 drugs (sensitivity = 0.669, 1-specificity = 0.423)], depression in the last 12 months (Figure 2b) [AUC = 0.835, 95% CI = 0.818–0.853; *p* < 0.001, the cut-off point was 2.5 drugs (sensitivity = 0.755, 1-specificity = 0.233)], anxiety in the last 12 months (Figure 2c) [AUC = 0.803, 95% CI = 0.784–0.821; *p* < 0.001, the cut-off point was 1.5 drugs (sensitivity = 0.867, 1-specificity = 0.415)] and multimorbidity (Figure 2d) [AUC = 0.825, 95% CI = 0.815–0.835; *p* < 0.001, the cut-off point was 1.5 drugs (sensitivity = 0.669, 1-specificity = 0.127)] predicted by the number of medications consumed in the last 2 weeks.

Table 2 shows the bivariate correlations and partial correlations between GHQ-12 score with MMS, the number of diseases (multimorbidity levels) and the number of medications consumed in the last 2 weeks. The GHQ-12 score was positively associated with the number of diseases and number of medications consumed. However, the GHQ-12 score was negatively associated with MMS. All of the correlations remained after adjusting for sex and age.

Table 3 shows the sociodemographic characteristics of the participants by level of PA. All variables were significant when related to PA levels. Pre-elderly who had a mental disorder such as anxiety or depression had lower levels of PA (*p* < 0.001). The average score in GH1-12 of people who had low levels of PA was 2.03 (DS ± 3.28) unlike the 0.88 (DS ± 1.87) of people who had high levels of PA (*p* < 0.001). Similarly, people with polypharmacy showed lower levels of PA (*p* < 0.001). In fact, the average number of drugs consumed by people who had low levels of PA was 2.09 (DS ± 2.03) unlike the 1.83 (DS ± 1.88) reported for people who had high levels of PA (*p* < 0.001).

**Figure 3:**
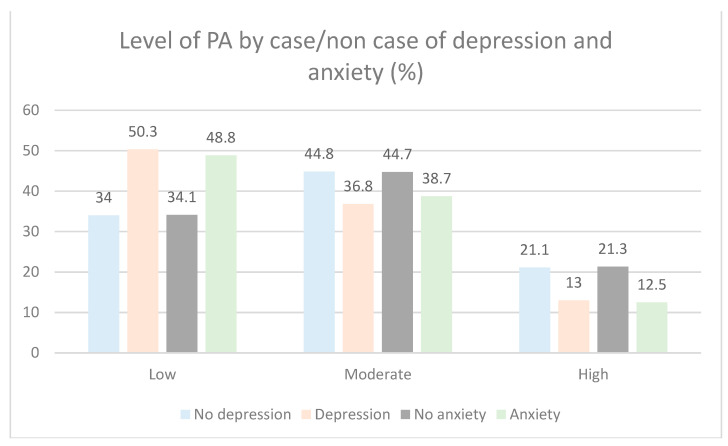
Distribution of levels of PA at depression and anxiety in Spanish pre-elderly (*n* = 5977).

Levels of PA and its relation to depression and anxiety can be seen in Figure 3 (*p* < 0.001).

In the adjusted logistic regression analysis (Table 4), we observed that low levels of PA were positively associated with being married (adjusted odds ratio = AOR: 1.26, 95% confidence interval = CI: 1.07–1.48, *p* = 0.005), without studies (AOR: 1.63, 95% CI: 1.26–2.11, *p* < 0.001), having anxiety (AOR: 1.28, 95% CI: 1.05–1.55, *p* = 0.014), having mental health vulnerability (AOR: 1.44, 95% CI: 1.18–1.76, *p* < 0.001), bad/very bad self-perception of their health (AOR: 2.1, 95% CI: 1.72–2.57, *p* < 0.001) and obesity (AOR: 2.07, 95% CI: 1.18–2.63, *p* = 0.011). In addition, in sex-stratified analyses (Table 5) among women, belonging to social class V and VI (AOR: 1.28, 95% CI: 1.02–1.61, *p =* 0.034) and having depression (AOR: 1.42, 95% CI: 1.1–1.84, *p* = 0.007) were positively associated with low levels of PA.

## 4. Discussion

To the best of our knowledge, this is the first study to analyze the relationship between the levels of PA, mental health conditions and the consumption of medications among the Spanish pre-elderly. The main findings were that low levels of PA were associated with mental health vulnerability, anxiety and depression (in case of women). In addition, people with low levels of PA consumed more medications than those with high levels of PA.

From a physiological point of view, the ageing process and advancing chronological age do not occur simultaneously. Ageing can be influenced by multiple factors in combination, including biological ageing, the occurrence of diseases and certain lifestyle patterns, such as a low level of PA [6]. Our results show that higher levels of PA are related to a lower prevalence of multimorbidity (no. of diseases), depression, anxiety and medication consumption. In line with another study carried out in Spain [45], it shows an inverse relationship between the consumption of certain medications for mental problems and levels of PA, and there may be a bidirectional relationship between these medications and PA. The results are in line with other studies indicating that PA confers considerable protection against chronic diseases (diabetes, hypertension and hypercholesterolaemia) as well as mental health problems such as anxiety and depression [3,4,20,21,46]. The practice of PA helps prevent depression where it is stated that performing flexibility exercises could reduce the risk of depression by up to 81% [47]. These results are in line with those obtained in the present study where an inverse relationship between PA and depression can be observed. In addition, the negative relationship found between MMS and medication consumption confirms how regular PA practice could reduce drug consumption, as in other studies [22].

The highest percentage of medications consumed was for cholesterol, blood pressure and pain, the consumption of which can be reduced through PA. A systematic review of randomized clinical trials showed that PA is an intervention with few adverse effects that reduces pain, provides physical and mental health benefits and improves quality of life [48]. Therefore, it can be said that a high level of PA, in addition to improving diseases, could reduce the consumption of medications and is associated with better overall health [6,22,46,49].

The WHO recommends undertaking at least 150 min of moderate PA, 75 min of vigorous PA or a combination of both every week, which corresponds to a moderate level of PA in our study [50]. However, our results show that more than a third of our population did not achieve these levels. This is a problem because PA can reduce the symptoms of depression and anxiety [49,50], a current issue in our society. This age range is important because it is on the verge of aging; therefore, the PA habits at this age are those that precede aging. In our study, it has been observed that PA levels are not met in this age range, as in the study by Biertnat et al. [25], where it was shown that people in this age range and retirees are the ones who undertake less PA. In our study, moderate levels of PA were lower than other study carried out with older people in Spain [41]. Moderate levels of PA should become a common habit in order to break the vicious circle of a sedentary lifestyle, obesity, depression, anxiety and incapacity [6]. Adequate levels of PA may improve mental health status [51]. According to the results obtained in the present study, it has been shown that PA has a positive influence on the prevention of depression, showing that if adults of this age range performed at least 1 h of exercise a week, new cases of depression could be prevented by up to 12% [52]. To achieve this, several systematic reviews show that eHealth interventions (such as interactive websites, smartphone applications, etc.) are effective in increasing PA levels in adults aged over 50 years [53,54]. The barriers that have been found to promote active aging have been described as a lack of time, organizational restrictions, limitations of being referred to adequate services, a lack of interest and support in prevention by administrators, a lack of incentives to participate, a lack of financial resources, too few personnel, and a lack of transportation, among others [5].

Furthermore, in line with previous studies, our results show that low levels of PA are related to being married [55], having a low educational level [14,24,56], anxiety [6,17], depression (in women) [6,14], low social class (in women) [55], worse self-perception of health status [14,21] and obesity [24,55]. In fact, a low educational level accompanied by a low socio-economic status (social classes V and VI) has been associated with a higher prevalence of obesity [24,55]. Therefore, understanding these factors is of particular interest when designing health policies aimed at promoting PA, especially in people with a lower level of PA.

Our results show a strong association between the number of diseases and drug consumption, which might seem obvious [23]. Nevertheless, in our logistic adjusted regression model, multimorbidity and disease or chronic/long-term illnesses were not associated with low levels of PA. Other studies have shown that physical function is related to the development and worsening of multimorbidity over time [57]. In high-income countries, the greatest increase in the prevalence of multimorbidity is recorded in two periods: between 50 and 60 years of age, and in old age [35], coinciding with the age of our participants. However, in agreement with another study, it appears that chronic disease is not the reason for the lower levels of PA observed [14,58]. On the contrary, self-perception of health status plays a more important role in low levels of PA than the mere presence of disease [14].

Finally, this study has some limitations. First, as it was a cross-sectional study, it was not possible to determine the causal direction between the associations found. Second, the measurement of PA levels and anthropometric data are self-reported and without objective measures. However, the present study has great strengths because the data were obtained from a national representative survey with a large number of participants and represents a step towards gaining knowledge of this problem in today’s society.

## 5. Conclusions

In conclusion, the Spanish pre-elderly do not reach the levels of PA recommended by the WHO. Low levels of PA were associated with being married, low educational levels, mental health vulnerability, anxiety and depression (in women), poor self-perception of health and low social class (in women). In addition, people with a low level of PA consumed more medications and had higher multimorbidity. The GHQ-12 score was positively associated with the number of diseases and number of medications consumed, and GHQ-12 score was negatively associated with MMS.

It is important that people reach old age with an optimal health status in order to reduce age-related disability and morbidity; this is possible with adequate levels of PA from earlier ages, such as pre-aging. Knowing the variables associated with low levels of PA allows us to direct health policies towards this population in order to improve their PA levels. Taking into account these variables, PA programs should be incorporated in the pre-elderly stage as this can prevent disease, reduce the consumption of medication and improve mental health, thus reducing visits to health services and the healthcare expenditure associated with the ageing of the population.

## Figures and Tables

**Table 1:** Sociodemographic characteristics of Spanish pre-elderly (age between 50 and 64 years old) (*n* = 5977).

	Men *n* = 2874 (%)	Women *n* = 3103 (%)	Total *n* = 5977 (%)	*p*-Value χ^2^
*Sociodemographic characteristics*				
*Marital status*				<0.001
Single	503 (17.5)	399 (12.9)	902 (15.1)	
Married	1976 (68.8)	1994 (64.3)	3970 (66.4)	
Widowed	62 (2.2)	288 (9.3)	350 (5.9)	
Separated	114 (4)	123 (4)	237 (4)	
Divorced	213 (7.4)	287 (9.2)	500 (8.4)	
Not responding	6 (0.1)	12 (0.3)	18 (0.2)	
*Level of education*				0.024
Without studies	171 (5.9)	208 (6.7)	379 (6.3)	
Primary	1395 (48.5)	1448 (46.7)	2843 (47.6)	
Secondary or PT	838 (29.2)	858 (27.7)	1696 (28.4)	
University	470 (16.4)	589 (19)	1.059 (17.7)	
*Social class*				<0.001
Class I	277 (9.6)	309 (10)	586 (9.8)	
Class II	204 (7.2)	239 (7.7)	443 (7.4)	
Class III	548 (19.1)	631 (20.3)	1179 (19.7)	
Class IV	490 (17)	368 (11.9)	858 (14.4)	
Class V	960 (33.4)	969 (31.2)	1929 (32.3)	
Class VI	375 (13)	505 (16.3)	880 (14.7)	
Not responding	20 (0.7)	82 (2.6)	102 (1.7)	
*Mental health variables*				
*Depression*				<0.001
No	2710 (94.3)	2704 (87.1)	2414 (90.6)	
Yes	164 (5.7)	399 (12.9)	563 (9.4)	
*Anxiety*				<0.001
No	2680 (93.2)	2697 (86.9)	5377 (90)	
Yes	194 (6.8)	406 (13.1)	600 (10)	
*Mental health vulnerability*				<0.001
Non case (GHQ-12 ≤ 5)	2643 (92)	2735 (88.1)	5378 (90)	
Case (GHQ-12 ≥ 6)	231 (8)	368 (11.9)	599 (10)	
*Health-related variables*				
*Health self-perception*				0.004
Very good	380 (13.2)	397 (12.9)	777 (13)	
Good	1549 (53.9)	1549 (49.9)	3098 (51.8)	
Regular	680 (23.7)	814 (26.2)	1494 (25)	
Bad	207 (7.2)	352 (8.1)	459 (7.7)	
Very bad	58 (2)	91 (2.9)	149 (2.5)	
*Disease or chronic/long-term illnesses*				<0.001
No	801 (27.9)	714 (23)	1515 (25.4)	
Yes	2073 (72.1)	2.388 (77)	4461 (74.6)	
*Multimorbidity*				<0.001
No	1251 (43.5)	1057 (34.1)	2308 (38.6)	
Yes	1623 (56.5)	2046 (65.9)	3669 (61.4)	
*BMI*				<0.001
Underweight	11 (0.4)	56 (1.9)	67 (1.1)	
Normal weight	744 (25.9)	1338 (43.1)	2082 (34.8)	
Overweight	1440 (50.1)	1041 (33.5)	2481 (41.5)	
Obesity	620 (21.6)	541 (17.4)	1161 (19.4)	
Not responding	59 (2)	127 (4.1)	186 (3.2)	
*Polypharmacy*				<0.001
No	2646 (92.1)	2729 (87.9)	5375 (89.9)	
Yes	228 (7.9)	374 (12.1)	602 (10.1)	
*Consumption of tranquilizers/relaxants/sleeping pills*				<0.001
No	2575 (89.6)	2508 (80.8)	5083 (85)	
Yes	299 (10.4)	595 (19.2)	894 (15)	
*Consumption of antidepressants/stimulants*				<0.001
No	2744 (95.5)	2761 (89)	5505 (92.1)	
Yes	130 (4.5)	342 (11)	472 (7.9)	
*Level of PA*				<0.001
Low	1006 (35)	1118 (36)	2124 (35.5)	
Moderate	1181 (41.1)	1454 (46.9)	2635 (44.1)	
High	687 (23.9)	531 (17.1)	1.218 (20.4)	

PT, professional training; GHQ, Goldberg Health Questionnaire; BMI, Body mass index; PA, physical activity.

**Table 2:** Bivariate correlations between GHQ-12 score with MMS, the number of diseases and number of medications consumed in the last 2 weeks.

	Simple Correlation				Partial Correlation ^a^			
	GHQ-12 Score	MMS	No. Diseases	No. Medications Consumed	GHQ-12 Score	MMS	No. Diseases	No. Medications Consumed
GHQ-12 score	-	−0.36 *	0.4 **	0.391 **	-	−0.33 *	0.4 **	0.393 **
MMS		-	−0.026 *	−0.043 **		-	−0.016	−0.037 *
No. diseases			-	0.707 **			-	0.694 **

Data are presented in the correlation coefficient R. * *p* < 0.05, ** *p* < 0.001. Abbreviations: GHQ-12, Goldberg Health Questionnaire; MMS, (unit of measurement for metabolic rate)-minutes per week; No., number. ^a^ Adjusted for sex and age.

**Table 3:** Characteristics of Spanish pre-elderly (age between 50 and 64 years old) by level of physical activity (*n* = 5.977).

	Level of Physical Activity			*p*-Value χ^2^
	Low *n* (%)	Moderate *n* (%)	High *n* (%)	
*Sociodemographic characteristics*				
*Sex*				<0.001
Men	1006 (35)	1181 (41.1)	687 (23.9)	
Women	1118 (36)	1454 (46.9)	531 (17.1)	
*Marital status*				0.042
Single	297 (32.9)	444 (49.2)	161 (17.9)	
Married	1439 (36.2)	1711 (43.1)	820 (20.7)	
Widowed	126 (36)	161 (46)	63 (18)	
Separated	81 (34.2)	107 (45.1)	49 (20.7)	
Divorced	175 (35)	206 (41.2)	119 (23.8)	
*Level of education*				<0.001
Without studies	181 (47.8)	158 (41.7)	40 (10.6)	
Primary	1060 (37.3)	1213 (42.7)	570 (20)	
Secondary or PT	571 (33.7)	755 (44.5)	370 (21.8)	
University	312 (29.5)	509 (48.1)	238 (22.5)	
*Social class*				<0.001
Class I	169 (28.8)	277 (47.3)	140 (23.9)	
Class II	126 (28.4)	205 (46.3)	112 (25.3)	
Class III	410 (34.8)	515 (43.7)	254 (21.5)	
Class IV	316 (36.8)	373 (43.5)	169 (19.7)	
Class V	756 (39.2)	822 (42.6)	351 (18.2)	
Class VI	308 (35)	399 (45.3)	173 (19.7)	
*Mental health variables*				
*Depression*				<0.001
No	1841 (34)	2428 (44.9)	1145 (21.1)	
Yes	283 (50.2)	207 (36.8)	73 (13)	
*Anxiety*				<0.001
No	1831 (34)	2403 (44.7)	1143 (21.3)	
Yes	293 (48.8)	232 (38.7)	75 (12.5)	
*Mental health vulnerability*				<0.001
Non case (GHQ-12 ≤ 5)	1808 (33.6)	2409 (44.8)	1161 (21.6)	
Case (GHQ-12 ≥ 6)	316 (52.8)	226 (37.7)	57 (9.5)	
*Health-related variables*				
*Health self-perception*				<0.001
Very good	188 (24.2)	344 (44.3)	245 (31.5)	
Good	1015 (32.8)	1410 (45.5)	673 (21.7)	
Regular	582 (39)	670 (44.8)	242 (16.2)	
Bad	240 (52.3)	173 (37.7)	46 (10)	
Very bad	99 (66.4)	38 (25.5)	12 (8.1)	
*Disease or chronic/ long-term illnesses*				<0.001
No	474 (31.3)	655 (43.2)	386 (25.5)	
Yes	1650 (37)	1979 (44.4)	832 (18.6)	
*Multimorbidity*				<0.001
No	742 (32.2)	1011 (43.8)	555 (24)	
Yes	1382 (37.7)	1624 (44.3)	663 (18)	
*BMI*				<0.001
Underweight	19 (28.4)	36 (53.7)	12 (17.9)	
Normal weight	668 (32.1)	931 (44.7)	483 (23.2)	
Overweight	843 (34)	1097 (44.2)	541 (21.8)	
Obesity	504 (43.4)	496 (42.7)	161 (13.9)	
*Polypharmacy*				<0.001
No	1850 (34.4)	2375 (44.2)	1150 (21.4)	
Yes	274 (45.5)	260 (43.2)	68 (11.3)	
*Consumption of tranquilisers/relaxants/sleeping pills*				<0.001
No	1720 (33.8)	2261 (44.5)	1102 (21.7)	
Yes	404 (45.2)	374 (41.8)	116 (13)	
*Consumption of antidepressants/stimulants*				<0.001
No	1899 (34.5)	2451 (44.5)	1155 (21)	
Yes	225 (47.7)	184 (39)	63 (13.3)	

PT, professional training; GHQ, Goldberg Health Questionnaire; BMI, Body mass index.

**Table 4:** Logistic regression model for the association between the low level of physical activity and sociodemographic and health characteristics of Spanish pre-elderly.

Variable	Simple Logistic Regression		Multiple Logistic Regression *	
	Crude OR (95% CI)	*p*-Value	Adjusted OR (95% CI)	*p*-Value
*Marital status*				
Single	Reference		Reference	
Married	1.16 (0.99–1.35)	0.06	1.26 (1.07–1.48)	0.005
Widowed/separated/ divorced	1.1 (0.92–1.33)	0.329	1.1 (0.9–1.34)	0.329
*Level of education*				
Without studies	2.19 (1.72–2.79)	<0.001	1.63 (1.26–2.11)	<0.001
Primary	1.42 (1.22–1.66)	<0.001	1.21 (1.03–1.42)	0.022
Secondary or PT	1.222 (1.03–1.44)	0.021	1.11 (0.94–1.32)	0.226
University	Reference		Reference	
*Anxiety*				
No	Reference		Reference	
Yes	1.85 (1.56–2.19)	<0.001	1.28 (1.05–1.55)	0.014
*Mental health vulnerability*				
Non case (GHQ-12 ≤ 5)	Reference		Reference	
Case (GHQ-12 ≥ 6)	2.2 (1.86–2.61)	<0.001	1.44 (1.18–1.76)	<0.001
*Health self-perception*				
Very good/good	Reference		Reference	
Regular	1.42 (1.25–1.6)	<0.001	1.21 (1.06–1.39)	0.004
Bad/very bad	2.8 (2.35–3.33)	<0.001	2.10 (1.72–2.57)	<0.001
*BMI*				
Underweight	Reference		Reference	
Normal weight	1.2 (0.7–2.05)	0.52	1.47 (0.84–2.56)	0.175
Overweight	1.3 (0.76–2.23)	0.339	1.53 (0.88–2.67)	0.131
Obesity	1.94 (1.13–3.39)	0.017	2.07 (1.18–3.63)	0.011

* R^2^ Nagelkerke value for multiple logistic regression = 0.050. Abbreviations: CI, confidence interval; OR, odds ratio; PT, professional training; GHQ, Goldberg Health Questionnaire; BMI, Body mass index.

**Table 5:** Logistic regression model for the association between the low level of physical activity and sociodemographic and health characteristics stratified by sex.

Variable	Men *		Women *	
	Adjusted OR (95% CI)	*p*-Value	Adjusted OR (95% CI)	*p*-Value
*Marital status*				
Single			Reference	
Married			1.5 (1.17–1.93)	0.002
Widowed/separated/ divorced			1.33 (0.99–1.76)	0.052
*Level of education*				
Without studies	1.68 (1.15–2.45)	0.007		
Primary	1.32 (1.04–1.67)	0.021		
Secondary or PT	1.16 (0.9–1.49)	0.254		
University	Reference			
*Social class*				
Class I and II			Reference	
Class III and IV			1.26 (0.99–1.59)	0.057
Class V and VI			1.28 (1.02–1.61)	0.034
*Depression*				
No			Reference	
Yes			1.42 (1.1–1.84)	0.007
*Anxiety*				
No				
Yes				
*Mental health vulnerability*				
Non case (GHQ-12 ≤ 5)	Reference		Reference	
Case (GHQ-12 ≥ 6)	1.35 (0.99–1.83)	0.052	1.43 (1.1–1.86)	0.01
*Health self-perception*				
Very good/good	Reference		Reference	
Regular	1.16 (0.96–1.4)	0.131	1.24 (1.03–1.5)	0.026
Bad/very bad	2 (1.5–2.68)	<0.001	2.17 (1.65–2.86)	<0.001
*BMI*				
Underweight	0.78 (0.23–2.67)	0.697	Reference	
Normal weight	0.64 (0.516–0.81)	<0.001	1.8 (0.94–3.47)	0.077
Overweight	0.72 (0.59–0.88)	0.001	1.78 (0.93–3.44)	0.084
Obesity	Reference	0.001	2.47 (1.27–4.8)	0.008

* R^2^ Nagelkerke values: Men Logistic Model = 0.037 and Women Logistic model = 0.064. Abbreviations: CI, confidence interval; OR, odds ratio; PT, professional training; GHQ, Goldberg Health Questionnaire; BMI, Body mass index.

## Data Availability

Publicly available datasets were analyzed in this study. This data can be found here: https://www.ine.es/dyngs/INEbase/es/operacion.htm?c=Estadistica_C&cid=1254736176783&menu=resultados&secc=1254736195295&idp=1254735573175#!tabs-1254736195295.
